# Development of a Rotation-Robust PPG Sensor for a Smart Ring

**DOI:** 10.3390/s25206326

**Published:** 2025-10-13

**Authors:** Min Wang, Wenqi Shi, Jianyu Zhang, Jiarong Chen, Qingliang Lin, Cheng Chen, Guoxing Wang

**Affiliations:** 1 School of Integrated Circuits (School of Information Science and Electronic Engineering), Shanghai Jiao Tong University, Shanghai 200240, China; 2 Shenzhen Ninenovo Technology Limited, Shenzhen 518000, China

**Keywords:** wearable sensors, smart ring, photoplethysmography (PPG), rotation-robust, LED-PD angle, Monte Carlo simulation

## Abstract

Cardiovascular disease (CVD) remains the leading cause of global mortality, highlighting the need for continuous vital sign monitoring. Photoplethysmography (PPG) is well suited for wearable devices. Smart rings, benefiting from dense capillary distribution and minimal tissue interference, can capture high-quality PPG signals with comfort, making them a promising next-generation wearable. However, ring rotation relative to the finger alters the optical path, especially for multi-wavelength light, thus reducing accuracy. This paper proposes a rotation-robust PPG sensor for smart rings. Monte Carlo simulations analyze photon transmission under different LED–photodiode (PD) angles, showing that at ±60°, green, red, and infrared light achieve optimal penetration into the microcirculation layer. Considering non-ideal conditions, the green-light angle is adjusted to ±30°, and a symmetrical sensor design is adopted. A prototype smart ring is developed, capable of recording 4-channel PPG, 3-axis acceleration, and 4-channel temperature signals at 100, 25, and 0.2 Hz, respectively. The system achieves reliable PPG acquisition with only 0.59 mA average current consumption. In continuous testing, heart rate estimation reached mean absolute errors of 0.82, 0.79, and 0.78 bpm for green, red, and IR light. The results provide a reference for future smart ring development.

## 1. Introduction

Cardiovascular disease (CVD), as the leading global cause of death, claims approximately 17.9 million lives annually, imposing substantial medical and economic burdens. Wearable technology offers a viable solution for continuous vital sign monitoring, enabling proactive interventions [1]. Overnight monitoring is particularly critical for detecting subtle changes, such as nocturnal hypertension [2]. Sleep disorders, strongly linked to CVD, can largely increase risk and prevalence [3], driven by rising psychosocial stress, further exacerbates CVD-related morbidity and mortality. Thus, developing various wearable devices [4] for long-term monitoring is essential to advancing prevention-focused healthcare, instead of the intermittent hospital monitoring model, with a potential risk of missing data [5].

Photoplethysmography (PPG), as a low-cost optical technique for detecting blood volume changes, has become a cornerstone in wearable healthcare technology [6]. While its applications in wrist-worn devices have advanced significantly, the emergence of smart rings offers a more compact and comfortable alternative, especially for overnight monitoring [7]. The finger, with its denser capillary distribution compared to the wrist, allows smart rings to extract richer PPG signal features and improve application performance. Furthermore, the academic interest and market presence of smart rings are rapidly increasing [8,9,10,11], driven by their inherent advantages, such as enhanced wearability [12], discreet design, and improved signal fidelity. These strengths position smart rings as a promising candidate to lead the next wave of wearable technology innovations [13].

However, unlike stationary PPG sensors in traditional setups, the circular design of a smart ring can cause unintentional rotation relative to the finger. This rotation alters the photon’s propagation path through finger tissues, leading to substantial variations in light scattering, absorption, and refraction. Such changes critically affect the quality of PPG signals by introducing inconsistencies and artifacts. Since the accuracy of physiological parameter evaluation, such as heart rate (HR) [14], blood oxygen saturation [15], blood pressure [16], and sleep apnea [17], relies heavily on high-quality PPG signals, these distortions can significantly compromise the reliability of measurements. For instance, a reduced signal-to-noise ratio or uneven illumination of tissue regions due to rotation could lead to erroneous parameter extraction, ultimately undermining the precision of health monitoring systems.

Previous studies on PPG sensors have made significant breakthroughs in improving the light conversion efficiency of light-emitting diodes (LEDs) and photodiodes (PDs), and layout optimization [10,18,19]. However, to the best of the authors’ knowledge, few researchers have conducted specialized layout design studies to overcome signal quality degradation caused by ring rotation. This study highlights the profound impact of rotational effects and aims to address these challenges through simulation-based optimization. By using Monte Carlo simulations, the variation in photon transmission during rotation was analyzed across three commonly used wavelengths—green, red, and infrared light. The results confirmed that photon distribution in finger tissues undergoes substantial changes due to rotation, reinforcing the need for robust sensor designs. An optimal PPG sensor layout with an LED-PD angle of ±60° was identified, minimizing signal distortion across all three wavelengths. This approach lays the foundation for designing more reliable PPG-based smart rings capable of maintaining signal quality and ensuring accurate physiological assessments under real-world conditions.

Considering that the simulation is an ideal modeling, and the performance of real system will be affected by the non-ideality, a test platform is developed to conduct a further experiment in this paper. The experiment result indicates that the PPG signals can achieve optimal average quality and consistency when the LED-PD angle is set to ±30° for green light and ±60° for red and IR light. A rotation-robust PPG sensor layout is proposed based on the result. Subsequently, this paper presents a smart ring capable of sampling 4-ch PPG signals, 3-axis acceleration signals, and 4-ch temperature at frequencies of 100, 25, and 0.2 Hz, respectively. Due to the proposed rotation-robust PPG sensor, the developed smart ring can obtain more reliable PPG signals while the driving current of LEDs with duty cycle scheme is reduced to 179 μA. It helps the smart ring maintain an average current consumption of only 0.59 mA during real-time signal acquisition and wireless transmission.

## 2. Methods

### 2.1. Monte Carlo Simulation

**Figure 1:**
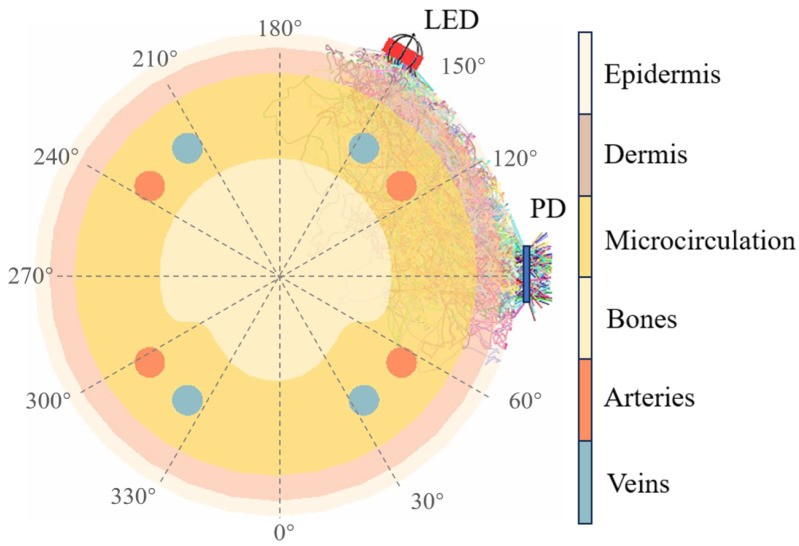
The cross-sectional diagram illustrates the propagation path of photons within the finger.

In order to enhance the rotational robustness of smart ring, a Monte Carlo simulation is used to find out the propagation path of photons through finger tissues and the impact of sensor rotation in this study. It is known that the optical phantom for the simulation can help in developing wearable devices [20]. The reflection, absorption, and scattering behaviors of photons in finger tissues are considered in this simulation. The finger model in Monte Carlo simulation [21] was established ideally based on anatomical reference literature. The total diameter of the finger is 19.2 mm, with a 0.6 mm thick epidermis on the surface, followed by a 1 mm thick dermis, and then a microcirculation with a diameter of 16 mm. Within the microcirculation are the arteries and veins with a diameter of about 1.2 mm and bones. Since there are individual differences in the distribution of capillaries, it is assumed that they are evenly distributed in the dermis and microcirculation layers. The cross-sectional view of the finger model is shown in Figure 1 using LightTools (version 2022.03).

The finger tissues have different optical parameters for lights of different wavelengths, so most commonly used wavelengths in PPG technology: 550 nm (Green), 628 nm (Red), and 940 nm (Infrared) are adopted in this simulation. The detailed optical parameters of finger tissues at specific wavelengths are based on previous research [22]. All simulations were performed on a high-performance computing workstation with the following specifications: Processor (CPU): Intel Core i9-13900K @ 3.00 GHz; Graphics Processor (GPU): NVIDIA GeForce RTX 3090 (24 GB VRAM); Memory (RAM): 64 GB DDR5; Operating System: Windows 10. On this hardware configuration, a simulation for a single wavelength using 5 × 107 photons had an average computation time of approximately 90 min.

### 2.2. Test Platform

**Figure 2:**
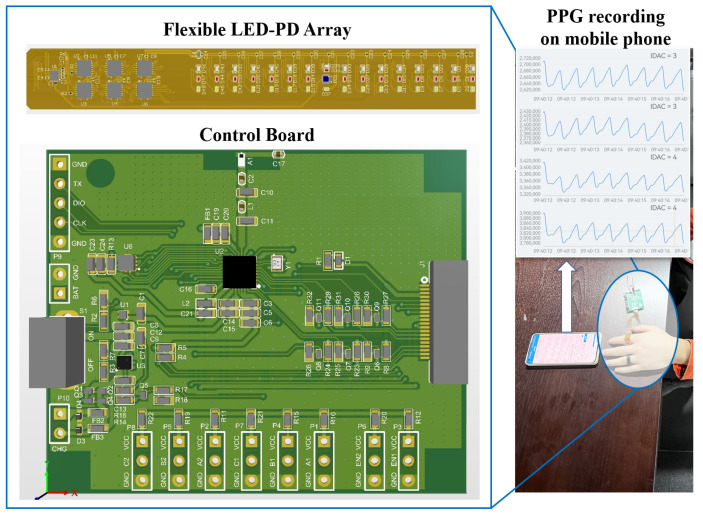
The test platform for the experiment to validate the PPG layout.

A test platform is designed for the experiment, as shown in Figure 2, which consists of a control board, a flexible LED-PD array, and the supporting software. Through the control board, the combinations of LED and PD can be selected. LEDs with wavelengths of 550, 628, and 940 nm, as in the simulation, are included. All the data were sampled with the sampling frequency (Fs) of 100 Hz, and transmitted to the supporting software through the Bluetooth Low Power (BLE) for recording and the signal processing. Informed consent was obtained from all participants.

To ensure the stability of the testing environment, data collection was conducted under indoor lighting to eliminate variations caused by ambient light. The room temperature is maintained at 22–25 °C to provide a comfortable testing environment. Additionally, participants were instructed to remain seated and avoid movement during the test to minimize motion artifacts in the signals. In this study, the left index finger was selected as the test object. Based on the simulation results, the finger was segmented into discrete regions with angular increments of 30°. A total of 10 subjects (6 males and 4 females, aged 22–45 years, body mass index from 17.6 to 30.4 kg/m^2^) participated in the experiment. Participants were of East Asian descent. The sizes of the rings were custom-made. For each subject, 432 sets of PPG signals were collected by combining different positions of the photodetector (PD) and light-emitting diode (LED), with each signal lasting for 60 s (144 sets corresponding to each of the three wavelengths).

### 2.3. Signal Quality Indices

For the test platform, it is difficult to evaluate the propagation paths of photons. Therefore, signal quality indices (SQIs) need be adopted in the physical experiment. There has been discussion about the SQIs of PPG signals [23,24,25]. In the past decades, the pulse wave amplitude (AC) and the perfusion index (PI) have been the common amplitude characteristics to assess signal quality [23]. The SNR indicates the ability to identify the weak changes in the signal [23]. Skewness and kurtosis of power spectral density are adopted to evaluate the distribution of the pulse wave over time [26]. In addition, PPG is a quasi-periodic signal whose periodicity affects the calculation of physiological index such as HR. Therefore, the maximum autocorrelation and the peak-to-peak interval corresponding to the autocorrelation function (AC) are used [27]. The performance of periodicity over time can be evaluated based on the standard deviation of the above two indicators (STDperiods and STDpeaks). To cover the amplitude, frequency-domain and time-domain features of PPG signals, a figure of merit (FoM) is defined as follows in Equation (1):

(1) $$FoM=\frac{PI\times AC\times S_{PSD}\times K_{PSD}}{STD_{periods}\times STD_{peaks}}$$

### 2.4. Signal Processing

The sampled PPG signals X are divided into N segments $x_{1},x_{2},\ldots ,x_{N}$ with fixed length of 6× Fs. A 4th-order Butterworth filter is employed to remove the baseline and high-frequency noise of segments. The cut-off frequency is set to [0.5, 5] Hz. The filtered segments are processed further as follows:

AC and PI: yi is divided into non-overlapping interval $w_{l}$ with the length of Fs. A local amplitude maxima and minima is applied on $w_{l}$ to detect the $w_{maxl}$ and $w_{minl}$ of the l-th interval. The AC value of each segment is computed as shown in Equation (2), where L is the number of the interval. The PI value is obtained from the ratio of ac to dc, as Equation (4), where the dc is extracted by computing the mean value of $x_{i}$, as Equation (3).

(2) $$AC=\frac{\sum_{l=0}^{L-1}\left(w_{maxl}-w_{minl}\right)}{L}$$

(3) $$DC=\frac{\sum_{i=0}^{N-1}x_{i}}{N}$$

(4) $$PI=\frac{AC}{DC}$$

SNR: There are many ways to define SNR. In this work, the SNR is defined as the ratio of signal power to its background noises, as shown in Equation (5), where $x_{highi}$ is the original signal $x_{i}$ passed through a high-pass filter with a cut-off frequency of 0.5 Hz, and $x_{lowi}$ is $x_{i}$ passed through a low-pass filter with a cut-off frequency of 5 Hz.

(5) $$SNR=10\times log(\frac{\sum_{t=1}^{T}\left(y_{i}^{2}\left[t\right]\right)}{\sum_{t=1}^{T}{\left(y_{i}\left[t\right]-x_{highi}\left[t\right]\right)}^{2}-\sum_{t=1}^{T}{\left(y_{i}\left[t\right]-x_{lowi}\left[t\right]\right)}^{2}})$$

**$S_{PSD}$** and $K_{PSD}$: The calculation of skewness and kurtosis is based on the literature [23]. f[nf] is the power value of the nf-th frequency component by applying FFT to yi, and $f_{mean}$, $f_{std}$, and NF are the mean value, the standard deviation, and the total number of frequency components, respectively.

(6) $$S_{PSD}=\sum_{nf=0}^{NF}{\left(\frac{f\left[nf\right]-f_{mean}}{f_{std}}\right)}^{3}$$

(7) $$K_{PSD}=\sum_{nf=0}^{NF}{\left(\frac{f\left[nf\right]-f_{mean}}{f_{std}}\right)}^{4}$$

$STD_{periods}$ and $STD_{peaks}$: The one-sided normalized AC [23] is applied. The m-th peak Rmax-m and the peak-to-peak interval Rppi-m will be detected from the autocorrelation signal R[k]i, where the autocorrelation signal can be calculated using Equation (8). STDperiods-i and STDpeaks-i can be obtained by computing the standard deviation of Rmax and Rppi separately.

(8) $$R{\left[k\right]}_{i}=\sum_{t=1}^{T}y_{i}\left[t+k\right]\times conj\left(y_{j}\left[t\right]\right)$$

After normalizing the computed indices, the FoM for each PPG segment can be calculated. PPG signals can provide multiple features, like the heart rate (HR). The detailed procedure for deriving heart rate (HR) from PPG signals is as follows: Firstly, the raw PPG signal collected by the ring sensor undergoes preprocessing: a 0.5–8 Hz band-pass filter is used to remove low-frequency baseline drift and high-frequency noise. Secondly, the feature extraction is performed: an adaptive threshold algorithm is used to identify the systolic peaks of the PPG waveform. Finally, the peaks are used to compute inter-beat intervals (IBIs), and the heart rate is calculated as 60 divided by the mean IBI.

## 3. Results

### 3.1. Finger Anatomy Analysis and Assumption

**Figure 3:**
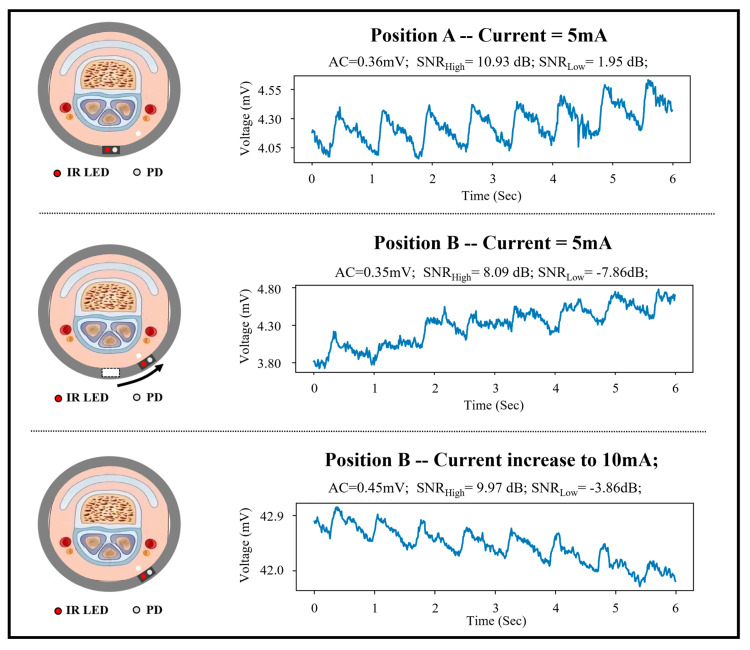
The impact of the rotation issue when smart ring during the real wearing scenarios. For example, the collected signals in position B are with bad signal quality.

Although some scholars have explored the optimal distances between LED and PD in reflective PPG through experiments or simulation analyses, the rotation influence in practical use was not considered. An actual test is conducted, as shown in Figure 3. The sensor can achieve signal-to-noise ratio (SNR) with a minimum value of 1.95 dB at the finger pulp, while the driving current of LED is 5 mA. However, when the sensor rotates at a certain angle, such as 30°, the SNR decreases to −7.86 dB, and the morphological features of signals are disrupted. Although doubling the driving current of LED to 10 mA can enhance the SNR to some extent, it cannot fully compensate for the impact of rotation and will increase power consumption. To address this issue, it is valuable to analyze the anatomical model of the fingers and conduct simulations based on it, thereby clarifying the impact of sensor rotation.

The finger is made up of several layers, starting from the outermost: epidermis, dermis, microcirculation with capillaries, adipose tissue, arteries, and bones. Each finger is supplied with four arteries, namely two digital volar proper arteries and two digital dorsal arteries, which are accompanied by nerves and are commonly located on the sides of the fingers. These arteries are commonly believed to be derived from changes in arterial blood flow. However, current studies have provided different perspectives. For example, a study [28] observed PPG signals from the palm area and reported a phenomenon that contradicts the assumption that PPG signals are directly caused by pulsatile variation of the arteries. In contrast, the capillaries contribute more to the PPG signals. Similarly, research found that PPG signals can be measured in bones and dental pulp [29]. The origin of the PPG signals is more likely to be due to the mechanical movements of the capillaries [30]. A study [31] also reported that the PPG signals of the green LED are highly correlated with the capillary distribution. It can be reasonably speculated that the periodicity of the PPG signals is due to the change in the optical properties of biological tissues caused by the mechanical motion of the capillaries. Therefore, this study assumes that the higher the proportion of photons passing through the microcirculation layer to the PD, the higher the quality of the PPG signals. An optimal PPG sensor layout with low sensitivity to rotation will be determined for different wavelengths on the basis of this assumption.

**Figure 4:**
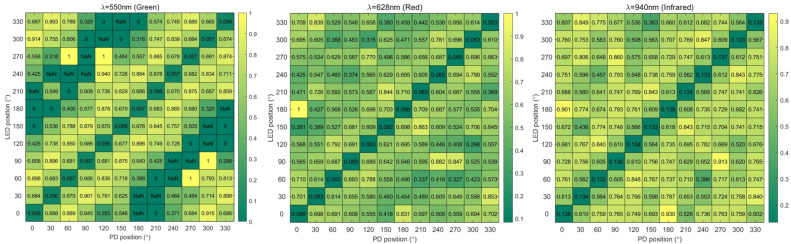
Monte Carlo simulation results indicate the proportion of photons passing through the microcirculation layer at different LEP-PD layouts.

The Monte Carlo simulation results are shown in Figure 4. NaN indicates that no photons are received by PD in simulation. Table 1 shows the results for different LED-PD angles across three wavelengths.

### 3.2. FoM Results

**Figure 5:**
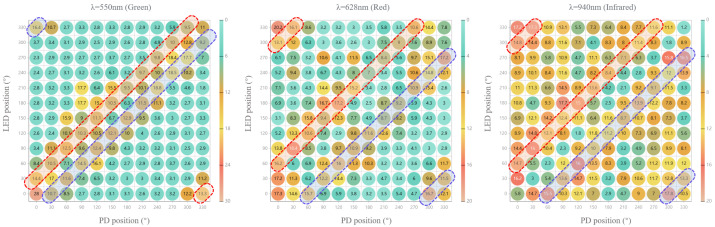
Heat maps showing the FoM results of the PPG signal received from each PD (x-axis) with each LED turned on (y-axis). The dashed box indicates the better results under different settings.

The heat maps display the FoM results of the green, red, and infrared light, respectively, as shown in Figure 5. The result shows a certain difference from the simulation, especially when the angle between LED and PD is 0°.

The FoM results are shown in Table 2. The quality of the PPG signal showing a good average FoM and consistency with the angle difference of ±30° for green light and ±60° for red and infrared light, respectively.

### 3.3. Performance Evaluation

**Figure 6:**
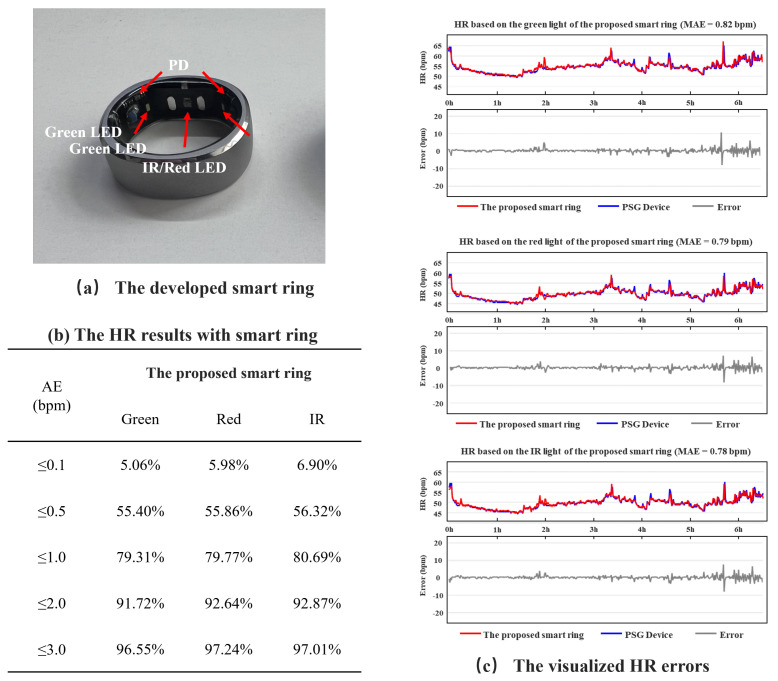
Robustness verification of PPG signal acquisition for the developed smart ring based on the proposed PPG sensor in this study.

To verify the improvement of the rotational robustness of the proposed PPG sensor and smart ring, a comparison experiment is conducted. A smart ring based on LED-PD angle of 0° is also developed as a reference in this study. The components, such as PPG sensor chip, remain the same for both smart rings and the driving current of LEDs is also consistent for both smart rings, which is 2 mA for green light and 8 mA for red/IR light. The robustness of PPG sensors is evaluated through the accuracy of HR calculated from the light with different wavelengths. The standard reference value for HR comes from polysomnography (PSG), the result can be found in Figure 6.

The hardware parameters of the developed smart ring system are shown in Table 3.

### 3.4. Design of the Smart Ring

**Figure 7:**
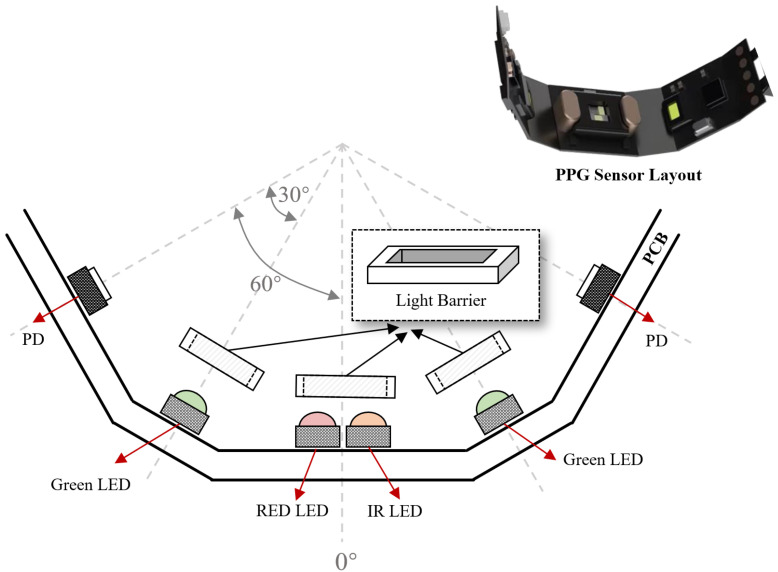
The proposed PPG sensor layout.

According to the simulation and experiment results, the PPG sensor layout for smart ring is designed as Figure 7 shown. The three wavelengths of light share PDs to reduce the numbers of devices. At the same time, the LEDs are surrounded by light barriers to prevent light to the PD directly, which reduces the impact on the DC component of PPG signal.

**Figure 8:**
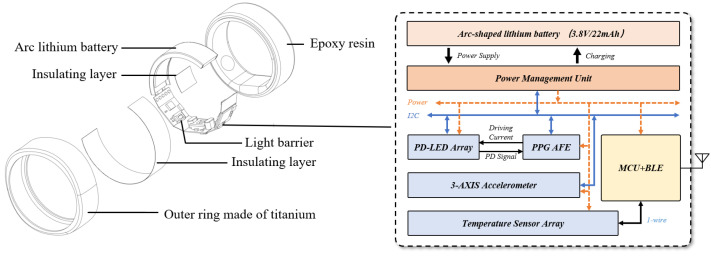
The architecture of the proposed smart ring.

Based on the PPG sensor layout, the overall smart ring is further designed, as shown in Figure 8. This work developed a complete hardware system, including structure and electronics. The outer ring of the system is made of titanium alloy to reduce the overall weight and thickness. All electronic components are designed on a complete flexible print circuit board (FPC). The FPC and battery are fixed in the outer ring by epoxy resin. The circuit is mainly composed of sensors, a power management part, a control, and a wireless transmission unit.

The PPG module is the most important sensor in the entire system. In addition to the layout design of the sensors, the timing control of the PPG module should also be carefully designed. As LEDs are the most power-hungry devices among sensors, the LED ON time has a significant impact on the average current consumption during signal acquisition.

**Figure 9:**
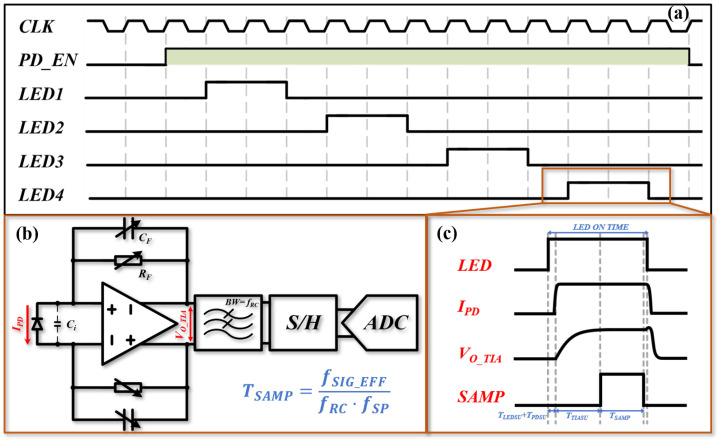
(**a**) The time diagram of the proposed PPG sampling; (**b**) TIA; (**c**) LED ON time.

To address this issue, a sampling strategy based on time division multiplexing, shown in Figure 9a, was adopted. Meanwhile, it is necessary to minimize the LED ON time without jeopardizing signal quality. In order not to stress the analog front-end circuit displayed in Figure 9b, the LED ON time should cover the sampling time of the analog-to-digital converter (ADC) (TSAMP) while meeting the bandwidth requirement of the transimpedance amplifier (TIA) (TTIASU), which is demonstrated in Figure 9c. In a typical PPG acquisition system, the effective bandwidth of the signal chain $fSIG_{EFF}$ can be defined as fRC × fSP × TSAMP, where fRC is the corner frequency of the low-pass filter for noise suppressing, and fSP is the sample frequency of the system. To avoid any non-linear phase distortions that corrupt the original signal, $fSIG_{EFF}$ should be larger than 6 Hz. Therefore, the $fSIG_{EFF}$ is designed to be 7.5 Hz with a sample frequency fSP of 100 Hz, leaving 25% margin for any process variation. To limit out-of-band noise, the fSP is chosen to be 2.5 kHz, which is the minimum available value, and according to the above consideration, the optimized TSAMP can be determined as 30 μs.

The PPG signal amplitude could vary dramatically according to different scenarios. For example, since strong sunlight can penetrate the finger and is collected by PD in outdoor conditions, a smaller transimpedance gain (about 10 kΩ) should be applied to avoid saturation of TIA and ADC. However, when collecting PPG indoors, a larger gain (about 1 MΩ) is preferred, as it will increase the SNR while saving the power of the LEDs. To maintain the stability of the TIA, the frequency compensation capacitor CF should also be selected correspondingly. The optimal capacitance could be calculated on the basis of the following equation to avoid overcompensation or oscillation.

(9) $$C_{F}=\sqrt{\frac{C_{i}}{2\pi R_{F}UGB}}$$

where $C_{i}$ is the total input capacitance of the TIA, which is dominated by the parasite capacitance of the PD. The UGB is the unit gain frequency of the operation amplifier. To match various RF settings, the CF needs to be adjusted between 20 and 2.5 pF, with corresponding values of Ci and UGB at approximately 20 pF and 1 MHz, respectively. In order to minimize quantization error arising from insufficient settling, it is recommended that TTIASU be at least 8 times larger than the time constant RFCF (2.5 μs), with a computed value of at least 20 μs.

Thanks to the advanced LED and PD technology, TLEDSU and TPDSU sum to a time delay less than 10 μs. Considering all the factors, the LED ON time is designed to be 70 μs with 10 μs margin for any LED ON/OFF damping, resulting in a duty cycle of 0.7% for each LED and reducing the power consumption dramatically.

Besides the PPG sensor, a low-power 3-axis accelerometer is employed to collect motion data to eliminate MAs. Related algorithm work has been presented in previous work [32]. Four temperature sensors with 0.0625 °C resolution are distributed on the PCB to establish a temperature gradient.

For the power management part, the whole system is powered by a 3.8 V/22 mAh arc-shaped lithium battery. Polarity correction circuit, programmable charger with I2C compatible serial interface, and low dropout regulator with 3.3 V output are designed in the power management unit (PMU). The PMU features a charging electrode connection without direction, and a built-in safe-timer preventing over-charging and preventing the host from running out of control, which ensures the security of the system. The control and wireless transmission unit of the system adopt a BLE wireless SoC with low communication power (4.1 mA TX at 0 dBm, 3.6 mA RX) and an Arm^®^ Cortex^®^-M33 core (27 μA/MHz active, 1.2 μA sleep).

### 3.5. Comparison

This study designs a smart ring with the proposed method and compares it with other works [8,9,10] to demonstrate its difference and advantages, as shown in Table 4. Radius and weight vary with different finger sizes. In this work, nine sizes were designed. The battery capacity is the same for each size.

## 4. Discussion

The great potential of PPG-based wearable devices are making significant contributions to preventive healthcare, and most devices are wrist-worn devices. However, the abundant static tissues located in the wrist, such as muscles and bones, will absorb the majority of photons and stimulate more noises. Therefore, the finger is a proper choice for collecting PPG signals [13].

In the simulation, the LED-PD angle is used to represent the specific PPG sensor layout. Different LED-PD combinations with the same LED-PD angle represent the rotation of the same PPG sensor layout in different positions. Considering the factor of actual manufacturing, the resolution of LED-PD angle is set to 30° in this simulation, as shown in Figure 2, resulting in 12 × 12 combinations (12 LED-PD angles and 12 positions for each LED-PD angle). The number of photons is set to 5 × 107. During the simulation, we found that photons mainly pass through the microcirculation layer, while the proportion of photons passing through arteries is less than 15%. This may be due to the small cross-section and deep location of the arteries in the fingers. Photons are more susceptible to the influence of capillary pulsations in microcirculation, which is consistent with the previous assumption. Therefore, in this study, we focus on the proportion of photons passing through the microcirculation layer.

The results are shown in Figure 4. NaN indicates that no photons are received by PD in simulation. According to the results, for instance, when the LED is at 270° and PD is at 60°, it will achieve the highest proportion of photons for green light. However, considering that rotation occurs in an actual scenario, the position of the PPG sensor will change randomly, and the LED and PD may rotate to 240° and 30°, respectively, which will cause a significant decrease in the proportion of photons. This explains why the rotation of the smart ring will affect the PPG signal quality. On the contrary, when using a PPG sensor layout with a LED-PD angle of 30°, it can achieve more stable results at various positions. Therefore, the performance of PPG sensor is evaluated by averaging the results of the combinations with the same LED-PD angle in this study.

Table 1 shows the results for different LED-PD angles across three wavelengths. It reveals that a specific LED-PD angle maximizes photon transmission through the microcirculation layer to the PD, with optimal angles around 60° for all three wavelengths. Additionally, the results are symmetrical, with angles like 60° and 300° yielding similar results, due to the symmetry of the finger anatomy. Green light (550 nm in this study) has a shallow penetration depth [30] in human skin, mainly interacting with the epidermis and superficial dermal capillaries (≤1 mm depth). Due to this limited penetration, a larger LED-detector angle (e.g., 60°) would cause most of the green light to scatter out of the detector’s field of view before interacting with capillary blood, leading to weak or noisy signals. A smaller angle (30°), however, shortens the horizontal distance between the LED and detector, ensuring that the green light—after shallow scattering in the upper skin layers—is more likely to be captured by the detector, thereby enhancing the signal-to-noise ratio (SNR) of the capillary blood volume fluctuation signal.

In contrast, red light (628 nm) and IR light (940 nm) have deeper penetration depths, reaching the deep dermal arterioles and venules. For these wavelengths, a larger angle (60°) is more advantageous: a wider LED-detector separation allows the light to cover a broader area of deep blood vessels, reducing the impact of local blood flow fluctuations (e.g., temporary constriction of a single superficial capillary) on the signal. This wider coverage enhances the stability of the PPG signal, which is critical for red/IR light’s primary application in measuring blood oxygen saturation (SpO2)—a metric requiring consistent detection of arterial blood flow. This wavelength-specific angle optimization is critical for the ring PPG sensor’s multi-wavelength performance, laying the foundation for the accurate, simultaneous monitoring of heart rate (dominated by green light) and SpO2 (dominated by red/IR light). This symmetry supports the design of a PPG sensor layout with two PDs sharing one LED, reducing components and power consumption. However, as the simulation is based on an assumed finger model, further experimental verification is still needed.

The heat maps display the FoM results of the green, red, and infrared light, respectively, as shown in Figure 5. The result shows a certain difference from the simulation. Especially when the angle between LED and PD is 0°. Except for non-ideal situations, the actual circuit cannot achieve complete overlap between PD and LED to obtain a 0° angle. Similarly, this study observes the results by categorizing them according to angles. The results are shown in Table 2. The quality of the PPG signal showing a good average FoM and consistency with the angle difference of ±30° for green light and ±60° for red and infrared light, respectively. In addition, the smart ring system is generally a symmetrical structure, and this angle difference still gives good results in symmetrical cases.

Figure 6 shows the results in the proposed PPG sensor and smart ring, the absolute error of HR based on green, red, and infrared light are 96.55%, 97.24%, and 97.01% within 3 bpm, respectively. Based on the prior experiment, it has been observed that the PPG sensor with LED-PD angle of 0° can yield high-quality signals for both green and red light when affixed to the finger pulp. However, during prolonged use, the smart ring is susceptible to rotation, leading to substantial variations in the signal quality captured by the sensor of this configuration. This is consistent with the results in Figure 9. The PPG sensor and smart ring proposed in this work can achieve continuous and stable HR monitoring throughout the night, with an overall mean absolute error (MAE) within 1 bpm, which reflects the robustness of the proposed smart ring system.

Further, Table 3 completes the performance evaluation. Due to the proposed PPG sensor for smart ring, the driving current of green, red, and IR LED can be maintained at 2, 8, and 8 mA, respectively. The green LED (2 mA drive) emits 0.239 mW/sr, while both the red and infrared LEDs (8 mA drive) emit 5.7 and 0.46 mW/sr, respectively. Combined with the low duty cycle, the total driving current can be as low as 179 μA. At such a low LED driving current cost, the system can still maintain stable PPG signal acquisition in practical scenarios. This helps the proposed smart ring only consume 0.59 mA, while 4-ch PPG signals, 3-axis accelerations, and 4-ch temperatures are sampled in 100, 25, and 0.2 Hz respectively, with the data being transmitting to a mobile phone in real time.

As shown in Table 4, the designed smart ring has advantages. Firstly, it is small, since the width and thickness is 7.8 and 2.6 mm. Secondly, it is lightweight and thus wearable. Thirdly, it is low power, the average power is 0.59 mA. Last but not least, the 4-channel PPG configuration (2G + 1R + 1IR) provides enhanced optical sensing capabilities compared to single-wavelength implementations in prior works [8,9,10].

Last, we acknowledge that beyond traditional PPG, several promising new technologies for pulsatile blood flow measurement and cardiac healthcare have emerged in recent years, including diffuse-optics-based method (DSPF) [35,36] and sound-based method [37,38]. In the future, it is necessary to focus on optimizing the design of wearable technologies, which represents the most prevalent technological path in both commercial and research domains.

## 5. Conclusions

Smart rings, thanks to inherent advantages of compact, insensitivity, and precision, have seen rapid development in recent years. However, due to the unique circular shape, rotation is a common occurrence during practical use, significantly impacting PPG signal quality. This study utilized Monte Carlo simulations to systematically reveal the extent to which smart ring rotation affects PPG signals. The results identified an LED-PD angle of 60° as optimal for green, red, and infrared light across various positions, providing valuable guidance for PPG sensor design. To fill the gap between simulation and real word, a practical testing system was developed, confirming that the optimal LED-PD angle is ±30° for green light and ±60° for red and infrared light, ensuring enhanced rotational robustness for smart ring designs. This finding serves as a valuable reference for the design of smart ring. Building on these findings, this study further designed and implemented a low-power rotation-robust smart ring. The average driving current required of four LEDs is only 179 μA, which is sufficient for obtaining reliable PPG signals during continuous monitoring, thereby effectively reducing system power consumption and enhancing signal stability. Additionally, the overall system power consumption is limited to 0.59 mA. With a typical 22 mAh arc-shape battery supply for smart rings, this system is expected to facilitate continuous data acquisition for over 30 h. Meanwhile, by enhancing rotational robustness, the proposed smart ring can achieve continuous HR measurement with MAE of 0.82, 0.79, and 0.78 bpm based on green, red, and IR light, respectively. This study proposes an effective PPG sensor layout for smart rings, combining Monte Carlo simulations and experiments. The design minimizes the impact of rotation during use without increasing LED current, offering valuable insights for enhancing robustness and achieving low-power smart ring designs. The simulation was constructed based on ideal conditions, only including key factors such as the angle of the LED detector and the basic light reflection characteristics; however, in actual experiments, complex real-world factors, such as the subtle differences in skin texture, the dynamic fluctuations of peripheral blood flow, and the instantaneous changes in the fit between the ring and the skin, could all have an impact on the optimal angle. Further research will further optimize the simulation model and gradually incorporate more real-world interfering factors to narrow this gap.

## Figures and Tables

**Table 1:** The average proportion of photons in the microcirculation layer at different LED-PD angles.

Δ	0°	30°	60°	90°	120°	150°	180°	210°	240°	270°	300°	330°
*λ* = 550 nm	0.057	0.680	0.878	0.707	0.517	NaN	NaN	NaN	0.483	0.707	0.876	0.680
*λ* = 628 nm	0.085	0.668	0.727	0.566	0.516	0.512	0.583	0.558	0.488	0.574	0.724	0.670
*λ* = 940 nm	0.135	0.594	0.784	0.751	0.658	0.648	0.649	0.685	0.694	0.744	0.782	0.595

**Table 2:** The FOM results of different angle differences between LED and PD (values shown as mean ± standard deviation).

Δ	0°	30°	60°	90°	120°	150°
*λ* = 550 nm	12.29 ± 6.2	13.86 ± 3.25	8.96 ± 3.39	3.74 ± 1.12	2.81 ± 0.42	2.92 ± 0.16
*λ* = 628 nm	7.26 ± 4.3	10.53 ± 4.41	13.03 ± 2.88	10.31 ± 3.15	5.82 ± 2.16	4.17 ± 2.00
*λ* = 940 nm	2.98 ± 1.60	11.22 ± 3.36	13.22 ± 2.97	11.51 ± 2.30	8.53 ± 3.05	7.12 ± 2.7
Δ	180°	210°	240°	270°	300°	330°
*λ* = 550 nm	3.00 ± 0.25	2.98 ± 0.37	4.18 ± 4.27	5.8 ± 5.31	9.17 ± 4.15	10.93 ± 1.61
*λ* = 628 nm	4.45 ± 2.21	3.69 ± 1.06	6.73 ± 3.36	10.05 ± 4.26	12.52 ± 3.96	8.57 ± 4.47
*λ* = 940 nm	7.76 ± 2.89	7.25 ± 2.8	9.83 ± 2.56	11.00 ± 3.76	12.87 ± 3.02	8.48 ± 3.54

**Table 3:** Current consumption of each module of the proposed smart ring.

Module	Consumption
Wireless Transmission	201 μA
4-channel PPG readout	157 μA (@100 Hz sampling rate)
LEDs driving current	179 μA (2 mA for green LEDs and 8 mA for Red/IR LEDs)
3-axis accelerometer	21 μA (@25 Hz sampling rate)
4-channel temperature	1.5 μA
Others	35 μA
Total	594.5 μA

**Table 4:** Hardware parameters comparison of smart ring devices. N.A. is short for non applicable.

Parameter	Device					
	This Work	[8]	[9]	[10]	[33]	[34]
Width	7.8 mm	22 mm	10 mm	7.9 mm	N.A.	11 mm
Thickness	2.6 mm	N.A.	2 mm	3.8 mm	1.6 mm	3.58 mm
Weight	3–5 g	N.A.	11 g	N.A.	2.5–2.8 g	4.3 g
Avg. Power	0.59 mA	3.76 mA	3 mA	2.5 mA	N.A.	40 mA
PPG Sensors	4	2	1	2	1	0
Accelerometer	3	3	0	0	9	0

## Data Availability

The datasets used and/or analyzed during the current study available from the corresponding author on reasonable request.
